# Development of Biphasic Injectable Hydrogels for Meniscus Scaffold from Photocrosslinked Glycidyl Methacrylate-Modified Poly(Vinyl Alcohol)/Glycidyl Methacrylate-Modified Silk Fibroin

**DOI:** 10.3390/polym16081093

**Published:** 2024-04-14

**Authors:** Rachasit Jeencham, Jiraporn Sinna, Chaiwat Ruksakulpiwat, Tulyapruek Tawonsawatruk, Piya-on Numpaisal, Yupaporn Ruksakulpiwat

**Affiliations:** 1 Research Center for Biocomposite Materials for Medical Industry and Agricultural and Food Industry, Nakhon Ratchasima 30000, Thailand; r_jeencham@sut.ac.th (R.J.); jiraporn.sinna@gmail.com (J.S.); charuk@sut.ac.th (C.R.); 2 Institute of Research and Development, Suranaree University of Technology, Nakhon Ratchasima 30000, Thailand; 3 School of Polymer Engineering, Institute of Engineering, Suranaree University of Technology, Nakhon Ratchasima 30000, Thailand; 4 Department of Orthopedics, Faculty of Medicine, Ramathibodi Hospital, Mahidol University, Bangkok 10400, Thailand; tulyapruek@gmail.com; 5 School of Orthopaedics, Institute of Medicine, Suranaree University of Technology, Nakhon Ratchasima 30000, Thailand

**Keywords:** poly(vinyl alcohol), silk fibroin, glycidyl methacrylate, biphasic injectable hydrogel, meniscus tissue engineering

## Abstract

The development of a hydrogel material with a modified chemical structure of poly(vinyl alcohol) (PVA) and silk fibroin (SF) using glycidyl methacrylate (GMA) (denoted as PVA-g-GMA and SF-g-GMA) is an innovative approach in the field of biomaterials and meniscus tissue engineering in this study. The PVA-g-GMA/SF-g-GMA hydrogel was fabricated using different ratios of PVA-g-GMA to SF-g-GMA: 100/0, 75/25, 50/50, 25/75, and 0/100 (*w*/*w* of dry substances), using lithium phenyl (2,4,6-trimethylbenzoyl)phosphinate (LAP) as a free radical photoinitiator, for 10 min at a low ultraviolet (UV) intensity (365 nm, 6 mW/cm^2^). The mechanical properties, morphology, pore size, and biodegradability of the PVA-g-GMA/SF-g-GMA hydrogel were investigated. Finally, for clinical application, human chondrocyte cell lines (HCPCs) were mixed into PVA-g-GMA/SF-g-GMA solutions and fabricated into hydrogel to study the viability of live and dead cells and gene expression. The results indicate that as the SF-g-GMA content increased, the compressive modulus of the PVA-g-GMA/SF-g-GMA hydrogel dropped from approximately 173 to 11 kPa. The degradation rates of PVA-g-GMA/SF-g-GMA 100/0, 75/25, and 50/50 reached up to 15.61%, 17.23%, and 18.93% in 4 months, respectively. In all PVA-g-GMA/SF-g-GMA conditions on day 7, chondrocyte cell vitality exceeded 80%. The PVA-g-GMA/SF-g-GMA 75:25 and 50:50 hydrogels hold promise as a biomimetic biphasic injectable hydrogel for encapsulated augmentation, offering advantages in terms of rapid photocurability, tunable mechanical properties, favorable biological responses, and controlled degradation.

## 1. Introduction

The meniscus, a C-shaped fibrocartilage structure located on the medial and lateral sides of the knee articular surface, plays a vital role as a shock absorber and load distributor [1,2]. Damage to the meniscus, often caused by excessive activities, sports, and accidents, can be challenging to repair due to the limited healing capacity of the white zone or inner avascular zone, similar to the articular cartilage [1,2,3]. This damage may eventually lead to osteoarthritis (OA), further complicating meniscus healing.

While meniscus repair procedures, particularly arthroscopic surgery, are considered less invasive with reduced infection rates [4], arthroscopic surgery has become a standard surgical technique [5,6]. However, current meniscus tissue engineering approaches require sophisticated techniques. In clinical practice, a simple cell-seeded scaffold method that can be utilized during arthroscopic surgery would be more practical [7,8].

Tissue engineering encompasses cells, bioactive molecules, and scaffolds. An ideal scaffold for cell growth must be biodegradable, biocompatible, and mechanically competent [8]. Biphasic injectable hydrogel technology offers a promising solution for meniscus tissue engineering by providing minimally invasive delivery, biological mimicry, biocompatibility, and personalized treatment options. These advantages position it as a significant tool in the development of effective therapies for meniscus injuries.

This injectable hydrogel comprises liquid and solid phases, where cells are initially seeded in a liquid (sol) and then encapsulated using a needle to form a solid hydrogel scaffold (gel) through photocrosslinking [9,10]. Hydrogels, 3-dimensional polymer networks with water-swelling and porosity properties, allow the homogeneous diffusion of solvents and nutrients [11]. Hydrogels can be fabricated in different forms depending on individual cell types. The hydrophilicity of hydrogels is particularly due to the presence of hydrophilic functional groups such as amide, carboxyl, amino, and hydroxyl groups distributed along the backbone of the polymeric chain [12,13]. Therefore, a cell-seeded biphasic injectable hydrogel as meniscus scaffold in arthroscopic surgery has been developed. Numerous researchers have studied the fabrication of injectable hydrogels, such as stearyl methacrylate/silk fibroin, genipin-crosslinked gelatin, and nano-hydroxyapatite/poly (L-glutamic acid)-dextran, for use in orthopedic and meniscus tissue engineering. Nevertheless, these hydrogels lack the required strength. Consequently, the development of a novel injectable hydrogel with excellent cell proliferation and high strength is crucial for applications of meniscus tissue engineering [14,15,16].

There are several materials used for fabricating hydrogels, including natural polymers and synthetic polymers. Poly(vinyl alcohol) (PVA) is a water-soluble synthetic polymer. The hydrophilic moieties provided by the hydroxyl group (-OH) on its backbone have made it a popular scaffold-supporting material for tissue engineering applications due to its non-toxicity, high mechanical properties, good biocompatibility, and biodegradability [17]. PVA hydrogels have limited intrinsic cell-adhesive properties, which can affect cell attachment, spreading, and proliferation within the scaffold [18]. Natural polymer-producing polymers have frequently been employed in tissue engineering applications because they are either components of or have macromolecular characteristics akin to the natural extracellular matrix (ECMs) [19]. Silk fibroin (SF), a natural polymer material that has been used as a suture in medicine for many years, is now widely recognized and used in many advanced biomedical applications [20], as it possesses biocompatibility, low immunogenicity, and anti-inflammatory properties, and promotes wound healing and chondrogenicity [21]. However, PVA and SF hydrogels generally offer low gel strength and water insolubility, which limits their applications to meniscus tissue engineering [22,23]. Their gel strength and water-insoluble properties can be improved by chemical structure modification (photocrosslinking) with the incorporation of a crosslinking agent and photoinitiator [24]. Photocrosslinked hydrogels are becoming increasingly important in biomedical applications because aqueous modified polymer solutions containing cells and bioactive substances can be supplied in a minimally invasive manner and crosslinked under physiological circumstances when exposed to ultraviolet light [24]. Controlling the intensity of the UV exposure and selecting an appropriate photoinitiator exposes the cells and bioactive molecules to the smallest number of unfavorable conditions [25]. In addition, the composite hydrogel from the combination of synthetic and natural polymers is one of the strategies to promote cell proliferation and appropriate mechanical properties.

Although there have been some studies investigating the development of injectable hydrogel systems based on PVA-g-GMA for various applications, it is important to note that high UV intensity and elevated concentrations of GMA can potentially be detrimental to cells [26,27]. Furthermore, despite these efforts, the reported PVA-g-GMA-based injectable hydrogels have not yet achieved the necessary mechanical properties for use in meniscus tissue engineering. Regarding the previously mentioned reasons, our study aims to develop an injectable hydrogel for meniscus scaffold from PVA-g-GMA and SF-g-GMA. The hydrogel materials were developed by modifying the chemical structures of poly(vinyl alcohol) (PVA) and silk fibroin (SF) using glycidyl methacrylate (GMA) (denoted as PVA-g-GMA and SF-g-GMA). The degree of methacrylate substitution on PVA-g-GMA and SF-g-GMA, modified with various amounts of GMA, was determined using Proton nuclear magnetic resonance (^1^H-NMR), and structural characterization was evaluated using Fourier transform infrared (FTIR) spectroscopy. In addition, the PVA-g-GMA/SF-g-GMA biphasic injectable hydrogel was formed by photocrosslinking with UV light. The PVA-g-GMA/SF-g-GMA biphasic hydrogel was investigated for its chemical interaction, morphology, pore size, mechanical properties, and biodegradability. Focusing on further applications in clinical practice, human chondrocyte cell lines (HCPCs) were seeded in the PVA-g-GMA/SF-g-GMA hydrogel to evaluate cellular compatibility (cell growth and cell viability) and chondrogenic gene expression.

## 2. Materials and Methods

### 2.1. Materials

Raw silk cocoons from the mulberry silkworm *Bombyx mori* were acquired from the Queen Sirikit Department of Sericulture Center in Nakhon Ratchasima, Thailand. Poly(vinyl alcohol) (PVA) (Mw 13,000–23,000, 87–89% hydrolyzed), glycidyl methacrylate (GMA, 97%), *N*,*N*,*N*′,*N*′-tetramethylethylenediamine (TEMED, 99%), and lithium phenyl(2,4,6-trimethylbenzoyl)phosphinate (LAP) were procured from Sigma-Aldrich Corporation (St. Louis, MI, USA). Anhydrous sodium carbonate (Na_2_CO_3_) and absolute anhydrous ethanol (EtOH) were obtained from Carlo Erba Reagenti (Rodano, Milan, Italy), and anhydrous calcium chloride (CaCl_2_) was sourced from ANaPURE (Auckland, New Zealand). Calcium nitrate 4-hydrate (Ca(NO_3_)_2_) was purchased from Kemaus. Dimethyl sulfoxide (DMSO) and acetone were procured from RCI Labscan Limited. Deuterium oxide (D_2_O, 99.9%) and dimethyl sulfoxide-*d*_6_ (DMSO-*d*_6_, 99.9%) were sourced from Cambridge Isotope Laboratories. SnakeSkin dialysis tubing (molecular weight cut-off, 10 kDa) was obtained from Thermo Fisher Scientific Inc. (Waltham, MA, USA).

### 2.2. Preparation of Glycidyl Methacrylate (GMA) Grafted onto Silk Fibroin (SF-g-GMA)

Raw silk cocoons were cut into small pieces and degummed with 1% (*w*/*v*) Na_2_CO_3_ at a weight ratio of 1:50, boiled at 98 ± 2 °C for 30 min to remove sericin, and then washed with deionized water several times. Subsequently, the degummed silk was dried overnight at 60 °C in a hot air oven. We dissolved 10 grams of degummed silk in 100 mL of a mixture of CaCl_2_/Ca(NO_3_)_2_/H_2_O/EtOH (30/5/45/20 weight ratio) in a microwave (Samsung, MS23K3513AW, 800 W). In the grafting process, 0.490 mmol of GMA was added to the SF solution. The mixture was stirred at 300 rpm at 60 ± 2 °C for 1 h. The mixture was then dialyzed with deionized water using SnakeSkin dialysis tubing, with a molecular weight cutoff of 10 kDa for 3 days, and deionized water was replaced every 4 h to remove salts and non-reactions. After completion of the dialysis, the undissolved impurities were removed by centrifugation at 10,000 rpm at 4 °C for 20 min to eliminate silk aggregates as well as debris from the original cocoons. The mixture was frozen at −60 °C for 12 h and freeze-dried at −70 °C for 48 h. The freeze-dried SF-g-GMA was stored at room temperature in a desiccator until further use.

### 2.3. Preparation of Glycidyl Methacrylate Grafted onto Poly(Vinyl Alcohol) (PVA-g-GMA)

To obtain a 5% (*w*/*v*) PVA solution, PVA was dissolved in DMSO and stirred at 60 °C until it became transparent. The catalysts, 100 mmol of GMA and 0.17 mL of TEMED were then added and stirred at 60 °C for 6 h and cooled to room temperature. The PVA-g-GMA solution was allowed to air dry in a fume hood for 24 h at the ambient temperature. Then, the PVA-g-GMA was dried in a 60 °C hot air oven for 48 h before transferring to a vacuum oven at 45 °C for another 24 h.

### 2.4. Nuclear Magnetic Resonance (NMR)

The ^1^H-NMR spectra of PVA, PVA-g-GMA, GMA, SF, and SF-g-GMA were characterized using nuclear magnetic resonance (NMR, Bruker, Avance III HD 500 MHz; Fällanden, Switzerland). For PVA, PVA-g-GMA, and GMA, 7 mg samples were dissolved in 500 μL of dimethyl sulfoxide-*d*_6_ (DMSO-*d*_6_) as the solvent. In contrast, 7 mg of SF-g-GMA, SF, and GMA were dissolved in 500 μL of deuterium oxide (D_2_O).

The degree of methacrylate substitution (DS%) of GMA grafted onto PVA was determined through ^1^H-NMR analysis, by calculating the relative area of the characteristic peaks of PVA, PVA-g-GMA, and GMA. Its value was assessed using the following Equation (1) [28]:

(1) $$\mathrm{DS}\%=\frac{(\mathrm{GMA}({\mathrm{CH}}_{2})/2)}{\mathrm{PVA}\left(\mathrm{OH}\right)+(\mathrm{GMA}({\mathrm{CH}}_{2})/2)}\times 100$$

The degree of methacrylate substitution (DM%) of GMA grafted onto SF-g-GMA was determined using the area of the proton peak of the aromatic ring in the tyrosine of SF (=6.7–7.3 ppm) and the proton peak of the vinyl group in GMA (=5.9–6.2 ppm), as follows in Equation (2) [29]:

(2) $$\mathrm{DM}\%=\frac{(\mathrm{GMA}({\mathrm{CH}}_{2})/2)}{\left(\mathrm{Tyrosine}/4\right)\times \;(\frac{100}{11.11})}\;\times \;\frac{100}{\mathrm{mole}\%\;\mathrm{of}\;\mathrm{reactive}\;\mathrm{group}}\times 100$$

Tyrosine was discovered to contain 11.11 mol% of the amino acids in SF after their composition was analyzed using an amino acid analyzer (L-8900, Hitachi High-Technologies Corporation, Tokyo, Japan). All samples were quantified using Bruker TopSpin software version 4.1.3.

### 2.5. Preparation of Glycidyl Methacrylate-Modified Poly(Vinyl Alcohol)/Glycidyl Methacrylate-Modified Silk Fibroin Hydrogel

The PVA-g-GMA (100 mmol of GMA) was dissolved in 10% *v*/*v* DMSO in deionized water to obtain a 10% (*w*/*v*) solution, stirred at 60 °C until a homogeneous solution was achieved. The 490 mM SF-g-GMA sponge was dissolved in deionized water at 50% *w*/*v* at room temperature. The mixed solutions of PVA-g-GMA and SF-g-GMA were thoroughly blended at different ratios of PVA-g-GMA to SF-g-GMA: 100/0, 75/25, 50/50, 25/75, and 0/100 (*w*/*w* of dry substances), followed by the addition of 0.3% (*w*/*v*) LAP. All gels were crosslinked with UV light (365 nm) for 10 min at an intensity of 6 mW/cm^2^. Molds were utilized to create a 96-well plate with a well diameter of 6.72 mm.

### 2.6. Fourier Transform Infrared Spectroscopy (FTIR)

The PVA-g-GMA/SF-g-GMA injectable hydrogels were frozen at −60 °C for 12 h and then freeze-dried at −70 °C for 48 h. A Bruker tensor Fourier-transform infrared spectrometer (FTIR, Bruker, Billerica, MA, USA) was utilized to characterize the functional groups of the hydrogels. Spectra were obtained in the wavenumber range of 4000 to 400 cm^−1^. Each run, conducted at a resolution of 4 cm^−1^, comprised 64 background scans and 64 sample scans. Every sample underwent three measurements, with each run taking place on a different section or side of the same sponge/hydrogel. To eliminate any residue from the previous sample, the attenuated total reflection (ATR) diamond crystal (TYPE A225/QL) was cleaned between samples using ethanol.

### 2.7. Gel Fraction

The PVA-g-GMA hydrogels were dried in a hot air oven at 40 °C for 24 h to a constant weight and weighed (Wo). The PVA-g-GMA hydrogels were immersed in deionized water for 24 h at 37 °C, then dried in a hot air oven at 40 °C for 24 h to a constant weight and weighed (Wf). The gel fraction was calculated according to the following equation:

(3) $$\mathrm{Gel}\;\mathrm{fraction}=\frac{\mathrm{Wf}}{\mathrm{Wo}}\times 100$$

### 2.8. Compression Test

The PVA-g-GMA/SF-g-GMA solutions were injected into a 96-well plate (6.72 mm in diameter and 2 mm in thickness) and crosslinked with UV light, followed by immersion in phosphate buffered saline (PBS) overnight. Compression testing of the PVA-g-GMA/SF-g-GMA hydrogels was conducted using a TA-XT plus texture analyzer (Texture Technologies Corp., London, UK) in compression mode, employing a 50 kg load cell. The PVA-g-GMA hydrogels were compressed at a constant rate of 5 mm/min, and three samples were tested for each group [30,31].

### 2.9. Morphological Structure Measurement

The PVA-g-GMA/SF-g-GMA hydrogel was frozen at −60 °C for 12 h and freeze-dried at −70 °C for 48 h. The samples were then examined for the morphologies using a field emission scanning electron microscope (FESEM; Carl Zeiss, Auriga, Oberkochen, Germany) at an operating voltage of 3 keV. The sample was coated with gold. The pore sizes were measured from 100 random pores from FESEM images in each sample using ImageJ software version 1.54h (Wayne Rasband NIH, Washington, DC, USA).

### 2.10. In Vitro Degradation

In vitro degradation of hydrogel in this study, which is intended for use in meniscus tissue engineering, involves simulating the conditions the hydrogel will encounter within the body over time.

The triplicate hydrogel samples were prepared in the same way as those for use for meniscus tissue engineering; they were fabricated in a 6.72 mm diameter mold with 2 mm thick. The samples were dried at 40 °C in a hot air oven for 24 h. They were then soaked in phosphate-buffered saline (PBS, pH = 7.4), a degradation medium that mimics the physiological conditions of the meniscus environment, and incubated at 37 °C with PBS solution refreshment in every 3 days. The biphasic hydrogels were washed with deionized water, dried, and weighed at 1, 2, 3, 4, 8, 12, and 16 weeks. The degradation rate was then calculated using Equation (4) [32], as follows:

(4) $$\%\;\mathrm{Residual}\;\mathrm{weight}=100-[(\frac{W_{f}}{W_{i}})\times 100]$$

where W_i_ is the initial dry weight of the sample, while W_f_ is the weight of the sample after immersing it in PBS.

### 2.11. Cell Viability and Live/Dead Cells in Three-Dimensional Cell Culture

The PVA-g-GMA/SF-g-GMA solutions were prepared at ratios of 100:0, 75:25, 50:50, 25:75, and 0:100. Subsequently, 0.3% *w*/*v* LAP photoinitiator was added to these solutions. Human chondrocyte cell lines (HCPCs) were obtained according to a protocol validated for purity, using flow cytometry analysis. The study was conducted following the guidelines of the Declaration of Helsinki and approved by the Institutional Review Board (Committee on Human Rights Related to Research Involving Human Subjects, Faculty of Medicine, Ramathibodi Hospital, MURA2022/469). HCPCs, at a density of 5 × 10^5^ cells/mL, were centrifuged in medium, and after medium removal, PVA-g-GMA/SF-g-GMA solutions were added to the cells at the same density. This mixture, referred to as a biphasic solution (PVA-g-GMA/SF-g-GMA biphasic hydrogel), was then injected into a 96-well plate (20 μL per well). UV photocrosslinking was performed on the solution mixtures for 10 min, followed by the addition of 100 μL of fresh medium. The cell-seeded photocrosslinked hydrogels were then incubated at 37 °C with 5% CO_2_ for 1, 3, and 7 days, with medium exchanged every 3 days. The survival rate of HCPCs in the biphasic hydrogel was assessed using a live and dead cell assay kit (Calcein AM, 7-AAD, ab270789) and Ethidium homodimer fluorometric detection reagent (ab145323). After 1, 3, and 7 days, the seeded HCPCs were stained and observed under a confocal microscope (Nikon ECLIPSE Ti). Green fluorescence indicated living cells, while red fluorescence demonstrated dead cells. The cell survival rate was calculated as the ratio of surviving cells (green) to total cells (green and red), counted using ImageJ software (Wayne Rasband NIH, Washington, DC, USA).

### 2.12. Gene Expression

A 20 μL biphasic solution, comprising PVA-g-GMA/SF-g-GMA solutions and HCPCs at a density of 5 × 10^5^ cells/mL, was injected into a 96-well plate, and the biphasic hydrogel was fabricated through UV photocrosslinking for 10 min. These hydrogels were cultured for 7, 14, and 28 days by using protocol according to [3]. Triplicate samples were applied in every hydrogel group. Total RNA was extracted from the HCPCs in the hydrogels using TRIzol reagent and the RNeasy mini kit (Qiagen, Hilden, Germany). Quantitative real-time polymerase chain reaction (qRT-PCR) was performed using the Fluorescein Kit (BIOLINE, London, UK) and the SYBR Green kit (Thermo Fisher Scientific, Waltham, MA, USA). The primer sequences for the qRT-PCR test are listed in Table 1.

### 2.13. Statistical Analysis

All parameters were calculated using triplicate sample sets for each experiment. General data were calculated using Microsoft Excel 2021 and reported as mean ± standard deviation (SD).

## 3. Results and Discussion

### 3.1. Synthesis of Glycidyl Methacrylate-Modified Poly(Vinyl Alcohol) and Glycidyl Methacrylate-Modified Silk Fibroin

**Figure 1:**
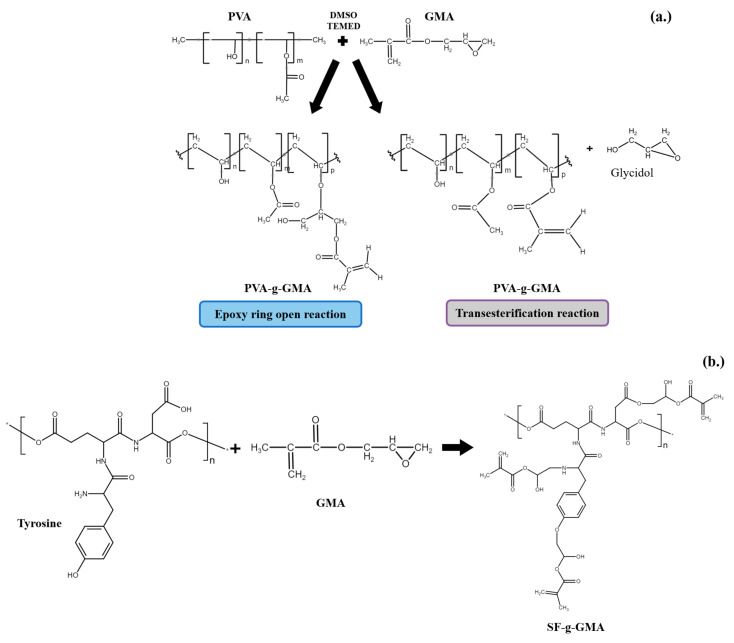

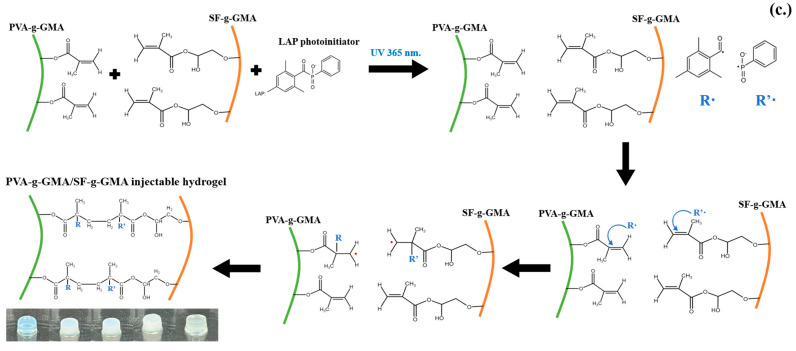
The schematic illustration of PVA-g-GMA (**a**), SF-g-GMA (**b**), and the PVA-g-GMA/SF-g-GMA hydrogel (**c**).

Figure 1 depicts the schematic illustration of PVA-g-GMA (a), SF-g-GMA (b), and the PVA-g-GMA/SF-g-GMA hydrogel (c). The grafting mechanisms of PVA and SF using GMA [28,29] were given in Figure 1a and b, respectively. In this study, the reaction mechanisms of PVA-g-GMA and SF-g-GMA was purposed based on NMR and FTIR results, as depicted in Figure 1c.

**Figure 2:**
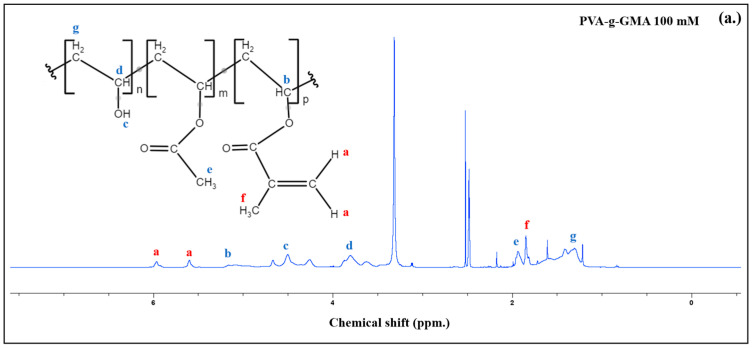

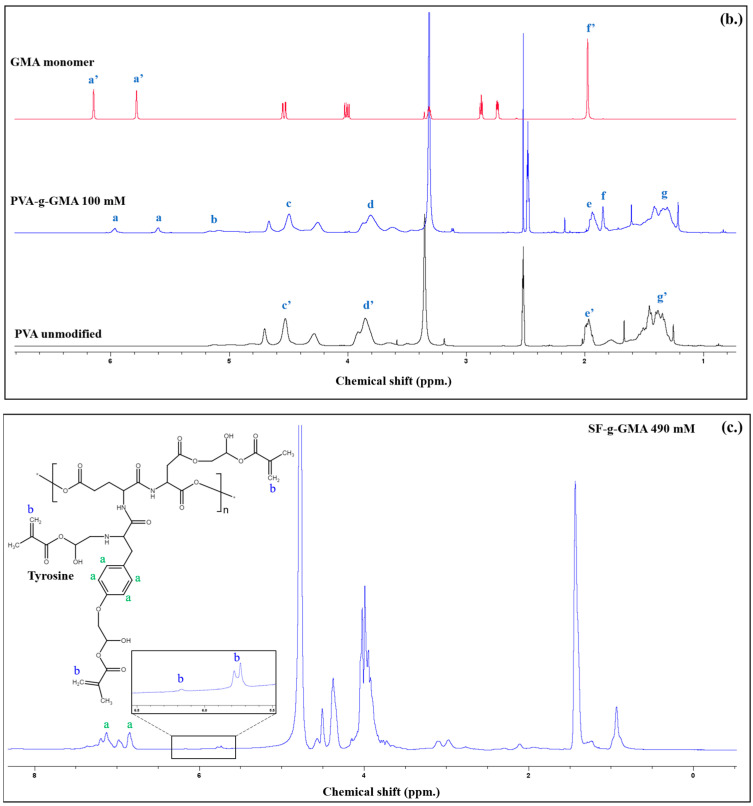
The molecular structure of PVA-g-GMA and ^1^H-NMR spectra (**a**), ^1^H-NMR spectra of GMA monomer, unmodified PVA, and PVA-g-GMA GMA contents at 100 mmol (**b**), and the molecular structure of SF-g-GMA 490 mmol and ^1^H-NMR spectra (**c**), The explanation of letters: a is (-OCO-C(CH_3_)=C**H_2_**), b is -CH- of O-methacrylate group, c is CH_2_-CH-O**H**, d is CH_2_-C**H**-OH, e is C**H_3_**-C=C, f is C**H_3_**-C=C, and g is C**H_2_**-CH-OH.

The methacrylate groups in PVA-g-GMA were demonstrated to confirm the transesterification reaction mechanism, which was determined using ^1^H-NMR spectra, as shown in Figure 2; the ^1^H-NMR spectra of unmodified PVA, GMA monomer, and PVA-g-GMA 100 mM are shown in Figure 1b. Sharp peaks at chemical shifts (δ) of 2.5 ppm were observed in the DMSO-*d*_6_ solvent in the polymer structures. The unmodified PVA spectra showed proton peaks of CH_2_-CH-O**H** that appeared at δ = 4.3–4.7 ppm, CH_2_-C**H**-OH at δ = 3.7–4.0 ppm, and C**H_2_**-CH-OH at δ = 1.4 ppm. New peaks appeared in the PVA-g-GMA spectrum at δ = 5.6 and 6.0 ppm, which corresponded to the protons of the vinyl group (-OCO-C(CH_3_)=C**H_2_**). These peaks were visible alongside the unmodified PVA peaks. These were ascribed to the characteristic double bond of the methacrylate group, verifying the successful grafting of the methacrylate group onto the pendant hydroxyl groups of the unmodified PVA. The methyl group (CH_3_-C=C) of the GMA unit was visible at δ = 1.9 ppm, while the CH group (-CH- of O-methacrylate group) of GMA was detected at δ = 5.2 ppm. The % GMA functional group, or the degree of methacrylate substitution (DS%) of the PVA-g-GMA 100 mM, was calculated using Equation (1). The DS% of GMA grafted on PVA was 12.06%.

GMA modified SF to enhance its water solubility and to determine the optimal %DM for improved solubility without compromising the SF properties. In the SF-g-GMA synthesis, 490 mmol of GMA was added to the SF solution, allowing GMA to react with amino groups (-NH_2_), hydroxyl groups (-OH), and carboxylic groups (-COOH). SF-g-GMA was synthesized by the ring-opening of the epoxy group in GMA. The modification of the SF side chain using GMA aimed to increase its water solubility. The ^1^H-NMR spectra of the GMA monomer, unmodified SF, and SF-g-GMA are presented in Figure 1c. The deuterium oxide (D_2_O) solvent exhibited a peak at δ = 4.7 ppm. In the unmodified SF ^1^H-NMR spectrum, the characteristic proton peak of the aromatic ring in tyrosine was at δ = 6.7–7.3 ppm, and lysine methylene was at δ = 2.65–3.05 ppm. Additionally, the SF-g-GMA spectra showed a subtle decrease in the tyrosine aromatic ring. New peaks indicated the creation of characteristic resonances of the methacrylate vinyl group at δ = 5.6–5.8 and 6.0–6.2 ppm, due to GMA inclusion. Tyrosine, constituting approximately 11.11 mol% of all amino acids, is an amino acid with reactive groups that GMA may modify [29]. With a GMA content of 490 mmol, the %DM of SF-g-GMA was 9.92%, possibly resulting from side reactions generated by the excessive addition of GMA molecules.

### 3.2. Fourier Transform Infrared Spectroscopy Analysis

**Figure 3:**
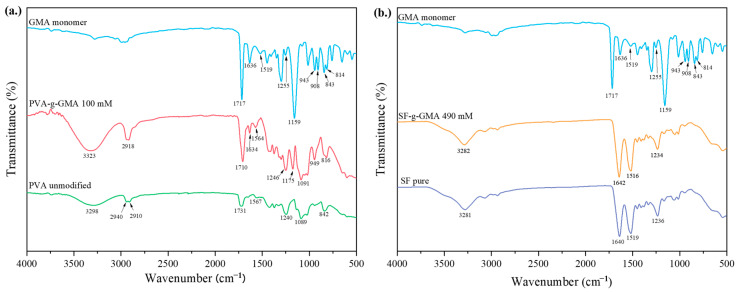
The FTIR spectra of unmodified PVA, GMA monomer, and PVA-g-GMA 100 mM (**a**) and the FTIR spectra of GMA, unmodified SF, and SF-g-GMA at 490 mM (**b**).

Figure 3a shows the FTIR spectra of unmodified PVA, GMA monomer, and PVA-g-GMA 100 mmol. Unmodified PVA exhibited transmittance bands at 842, 1089, 1567, 1707, 2940, 2910, and 3298 cm^−1^, representing =C-H stretching, C-O stretching, C=C stretching, C=O (ester group), CH_2_ symmetric stretching, C-H stretching, and O-H stretching, respectively. The GMA monomer showed peaks at 1255 cm^−1^ (breathing), 908 cm^−1^ (asymmetric deformation), and 843 cm^−1^ (symmetrical deformation), which are typical of epoxy groups. The carbonyl group (C=O) exhibited a strong peak at 1717 cm^−1^, followed by a C=C peak at 1636 cm^−1^ and a robust peak at 1159 cm^−1^, indicating C-O stretching of the ester group. The spectra of PVA-g-GMA revealed unique bands of PVA and GMA, such as C=O bending at 1710 cm^−1^ and C=C at 1634 cm^−1^, R_2_C=CH_2_ out-of-plane bending vibration at 949 cm^−1^, and C-O group stretching vibration at 1175 cm^−1^. Furthermore, the absence of bands for the GMA epoxy ring at 1255 cm^−1^ (breathing), 908 cm^−1^ (asymmetric deformation), and 843 cm^−1^ (symmetrical deformation) significantly indicates that transesterification was the mechanism of GMA and PVA grafting reaction.

In Figure 3b, the FTIR spectra of GMA, unmodified SF, and SF-g-GMA at 490 mM are presented. In the spectrum of unmodified SF, peaks at 1638 cm^−1^ (Amide I), 1517 cm^−1^ (Amide II), and 1234 cm^−1^ (Amide III), corresponding to random coil, β-sheet, and ω-helix, respectively, were observed. For SF-g-GMA at 490 mM, transmittance bands were at 1642 cm^−1^ (Amide I), indicating random coil, with a shift to 1638 cm^−1^ compared to unmodified SF; the wavenumber at 1514 cm^−1^ (Amide II) was assigned to β-sheet; and the wavenumber at 1234 cm^−1^ (Amide III) was assigned to α-helix. Small peaks at 949 and 1168 cm^−1^ on SF-g-GMA were attributed to the R_2_C=CH_2_ wagging stretching of the vinyl methacrylate group in GMA.

**Figure 4:**
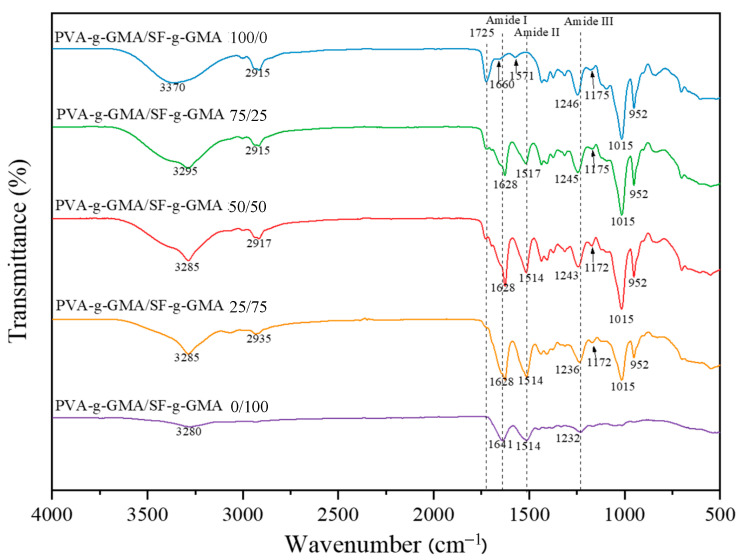
FTIR spectra of PVA-g-GMA/SF-g-GMA hydrogels.

The FTIR spectra of the PVA-g-GMA/SF-g-GMA hydrogel are illustrated in Figure 4. The primary characteristic peaks of the PVA-g-GMA hydrogel were observed at 2915 cm^−1^, 1725 cm^−1^, and 1660 cm^−1^, corresponding to CH_2_ (stretching vibration), C=O (stretching vibration), and (C=C), respectively. An intense hydroxyl (OH) band was observed at 3000–3600 cm^−1^, with a peak at 3370 cm^−1^ attributed to hydrogen bonds. Additionally, 1246 cm^−1^ corresponds to -C-O-C- stretching, with peaks at 1092 cm^−1^ attributed to CO stretching and OH bending, 947 cm^−1^ to bending -CH_2_, and 838 cm^−1^ to -CH rock. The primary characteristic absorption bands of the SF-g-GMA hydrogel are 1641 cm^−1^ (for amide I, C-O stretching, random coil/α-helix), 1514 cm^−1^ (for amide II, secondary N-H bonding, due to the β-sheet structure), and 1232 cm^−1^ (for amide III, N-H and C-N functionalities). The presence of a significant band of SF-g-GMA in the PVA-g-GMA hydrogel structure was confirmed by the change in the spectrum of the PVA-g-GMA/SF-g-GMA hydrogel on the amide I peak from 1641 cm^−1^ to 1628 cm^−1^, with an increase in SF-g-GMA content due to the polarity of the alcohol group (-OH) in PVA-g-GMA, inducing a conformational change in silk fibroin. This interaction led to the transition from random coil/α-helix to β-sheet conformation, resulting in a physical crosslink in the PVA-g-GMA/SF-g-GMA biphasic hydrogel [33,34].

The FTIR results indicated that the PVA-g-GMA/SF-g-GMA hydrogel had a more pronounced β-sheet structure than the pure SF sample (PVA-g-GMA/SF-g-GMA ratio at 0/100). The ratios of 75/25, 50/50, and 25/75 exhibited similar peaks [31], characterized by transmission bands typical of both components. Additionally, the OH region (3000–3600 cm^−1^) showed an extended width, suggesting a decrease in the intensity of hydrogen bonding. FTIR spectral results implied interactions between SF molecules and PVA [35].

### 3.3. Gel Fraction

**Figure 5:**
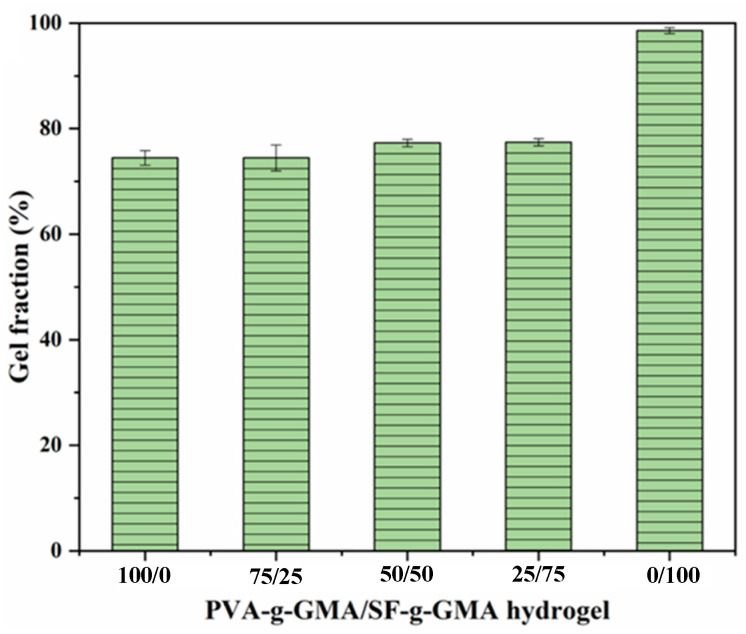
Gel fraction of PVA-g-GMA/SF-g-GMA hydrogels.

The gel fraction is the crosslinking degree of crosslinked hydrogel after water immersion. The percentage of undissolved fractions indicates the percentage of crosslinks formed. Typically, the gel fraction requirement of hydrogels should be ≥80% [36]. When the UV irradiates, the LAP absorbs the UV and converts this light energy into chemical energy, in the form of free radicals, which subsequently initiates crosslinking by polymerizing the methacrylate units of PVA-g-GMA or SF-g-GMA and forming a hydrogel. This indicates that prolonging the irradiation for an optimum time (10 min) could allow an increase in the number of free radicals, which may result in a high gel fraction (80–98%) or a high degree of crosslinking of the PVA-g-GMA/SF-g-GMA hydrogel. The gel fractions of PVA-g-GMA/SF-g-GMA hydrogels with different contents are shown in Figure 5. The PVA-g-GMA/SF-g-GMA hydrogel at ratios of 100/0, 75/25, 50/50, and 25/75 exhibits a nearly 80% gel fraction. In particular, the PVA-g-GMA/SF-g-GMA hydrogel at the ratio of 0/100 showed the largest gel fraction (98%) compared to other hydrogels. As previously studied, the polarity of the alcohol led to some degree of β-sheet crystallization, which encouraged physical crosslinks in the silk structure and led to increased gel formation. Therefore, a longer carbon chain means a lower polarity, which leads to a higher degree of physical crosslinking in the structure of the silk [37]. From the results, the gel fraction of all hydrogels met the requirement, implying that they are hydrophilic polymer networks that maintain their integrity in an aqueous environment.

### 3.4. Mechanical Properties of Glycidyl Methacrylate-Modified Poly(Vinyl Alcohol)/Glycidyl Methacrylate-Modified Silk Fibroin Hydrogel

**Figure 6:**
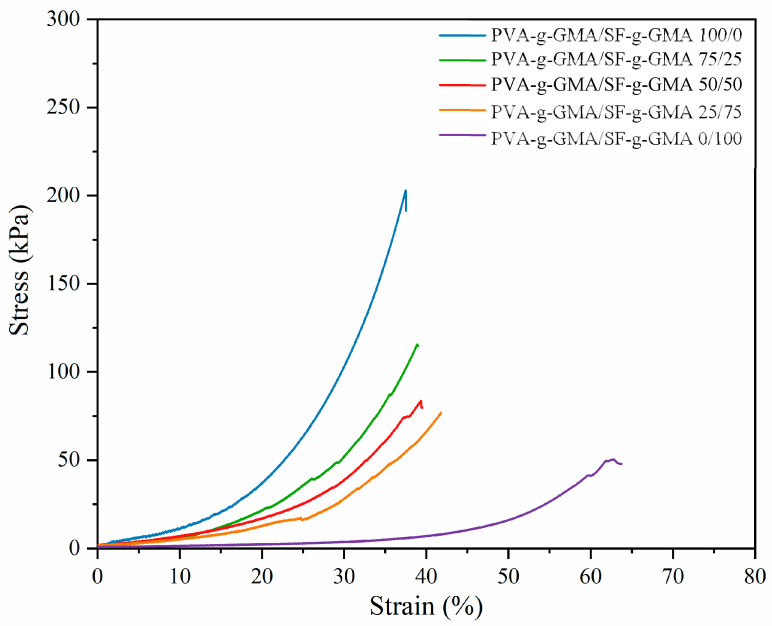
Stress–strain curve of PVA-g-GMA/SF-g-GMA hydrogel.

The mechanical properties of hydrogels play an important role in mimicking meniscus tissue [38]. The mechanical environment has a considerable influence on cellular functions, since cell adhesion and mechanical properties can impact cellular responses [39]. Generally, the compressive modulus of human meniscus is characterized by a range of 100–150 kPa [40]. In this study, the PVA-g-GMA/SF-g-GMA hydrogel was fabricated through photocrosslinking and evaluated under compression stimulation. The stress–strain curve is shown in Figure 6 and the mechanical properties of the PVA-g-GMA/SF-g-GMA injectable hydrogel are presented in Table 2. Martin Seitz A et al. reported that the 10–15% strain is in the range of physiological meniscal loading that occurs during daily life. Therefore, in this study, the compressive modulus was determined by calculating the slope of the initial linear region of the stress–strain curve (approximately 10% strain) [41]. The injectable hydrogel with SF-g-GMA contents of 0, 25, 50, 75, and 100% *w*/*w* showed a compressive modulus of 173, 146, 117, 93, and 11 kPa, respectively (Table 2). The compressive modulus of the PVA-g-GMA/SF-g-GMA injectable hydrogel decreased with increasing SF-g-GMA content. Despite this, the compressive modulus properties of the hydrogel closely resembled those of the human meniscus. This indicated that the PVA-g-GMA/SF-g-GMA hydrogel exhibited good mechanical properties while mimicking the mechanical characteristics of meniscus tissue.

While scaffolds with outstanding mechanical properties hold promise, they might not guarantee that the regenerated tissues will possess high mechanical qualities. Therefore, ongoing research is focusing on determining whether a biomimetic design would effectively provide a functional meniscus that is both mechanically and biologically developed. Future studies are anticipated to explore the relationship between in vivo settings and design-based functionalities.

### 3.5. The Morphology of Glycidyl Methacrylate-Modified Poly(Vinyl Alcohol)/Glycidyl Methacrylate-Modified Silk Fibroin Hydrogel

**Figure 7:**
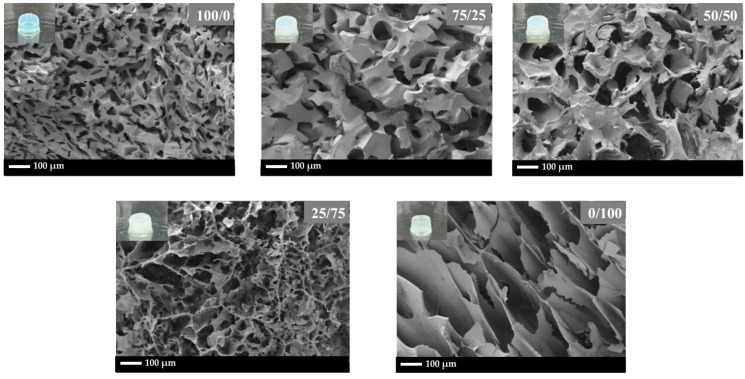
The SEM images of PVA-g-GMA/SF-g-GMA hydrogels with different ratios: 100/0, 75/25, 50/50, 25/75, and 0/100. The scale bar indicates 100 μm.

The morphology of the hydrogel cross-section, after complete swelling and freeze-drying, obtained using FESEM, is illustrated in Figure 7. The hydrogels exhibit a network structure characterized by high porosity.

The porosity and pore size of gels are dependent on a complex interplay of various parameters, such as polymer type and concentration, crosslinking density, solvent evaporation, and fabrication method, with each contributing to the overall microarchitecture and subsequently affecting the mechanical properties of the gel [42,43]. In this study, polymer type seems to play a dominant role in governing porosity and pore size. For PVA-g-GMA, the smallest pore size was obtained. This suggests that the molecular structure of PVA-g-GMA favors the formation of a more compact network with smaller void spaces. SF-g-GMA demonstrates a large pore size and thin wall thickness. With a decreasing SF-g-GMA fraction in PVA-g-GMA/SF-g-GMA, less porosity and smaller pore sizes, with a thicker wall thickness, were observed.

**Figure 8:**
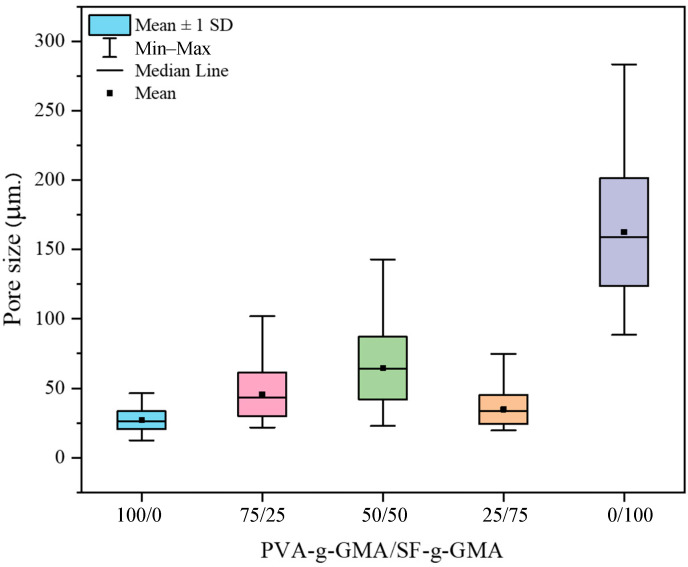
Pore size distribution of PVA-g-GMA/SF-g-GMA hydrogel.

The average pore size and pore size distribution of the PVA-g-GMA/SF-g-GMA hydrogel increased with increasing SF-g-GMA content, up to 50/50 (Figure 8). It should be noted that for PVA-g-GMA/SF-g-GMA at the ratio of 25/75, non-homogeneous porosity of hydrogels indicating by variations in pore size, distribution, or density within the hydrogel matrix was presented. Non-uniform porosity may result in uneven cell distribution within the hydrogel, affecting tissue formation and integration and also leads to mechanical weakness due to regions of lower density or larger pores exhibiting reduced mechanical strength. For the optimum pore size, pore sizes between 10 and 30 micrometers have been reported to have advantages for HCPCs [44]. However, the ideal hole size to promote the development and operation of HCPCs might change based on a number of variables, such as the intended tissue regeneration result, the particular application, and the stage of cell differentiation. The small pore size of PVA-g-GMA resulted in a high compression modulus, while SF-g-GMA, with a large pore size, exhibited the lowest compression modulus, as discussed in Section 3.4 (the mechanical properties of PVA-g-GMA/SF-g-GMA hydrogel).

### 3.6. In Vitro Degradation

**Figure 9:**
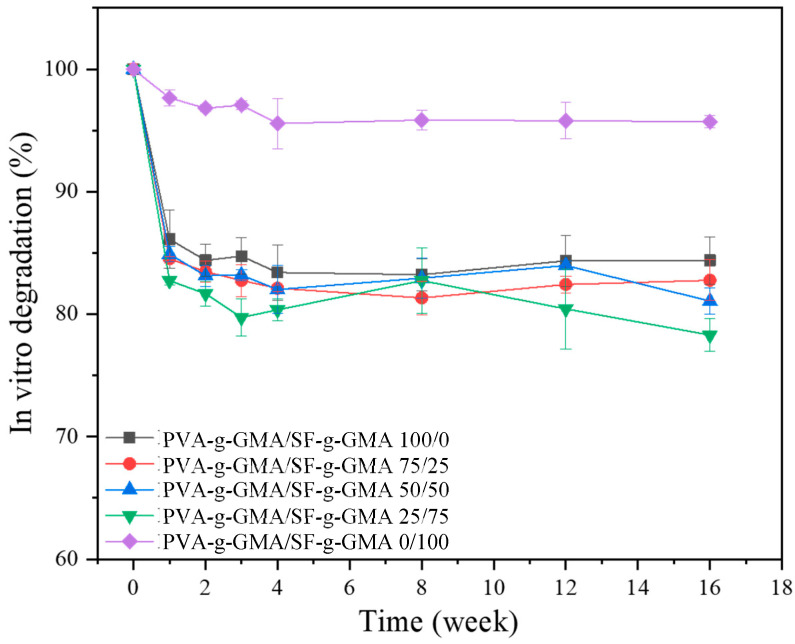
The graph demonstrated in vitro degradation of PVA-g-GMA/SF-g-GMA biphasic hydrogels in different ratios.

In this study, the in vitro degradation experiment was constructed by simulating the conditions the hydrogel will encounter within the meniscus environment over time after being injected into the body. The sharp decrease in weight loss during the first week was due to a rapid initial release of unreacted components (sol) from the hydrogel. However, this initial loss of material may not necessarily reflect the true degradation of the hydrogel structure but rather the leaching out of components that were not fully incorporated or crosslinked during the fabrication process. The rapid initial release of unreacted components from the hydrogel during the in vitro degradation experiment is indeed a critical aspect to consider and should be reported, especially in the context of arthroscopic-assisted surgery techniques, where understanding the behavior of the hydrogel post-injection is crucial for surgical planning and outcomes. After the first week, a gradual decrease in the hydrogel’s weight was noted (Figure 9). In 4 months, the degradation rates of PVA-g-GMA/SF-g-GMA at ratios of 100/0, 75/25, and 50/50 reached 15.61%, 17.23%, and 18.93%, respectively. The PVA-g-GMA/SF-g-GMA 25/75 construct showed the highest degradation rate, reaching 17.26% in 2 months and up to 21.70% in 4 months. The PVA-g-GMA/SF-g-GMA 0/100 hydrogel showed the lowest degradation rate of 4.30%. The PVA-g-GMA/SF-g-GMA hydrogel at the ratio of 0/100 exhibited the highest gel strength (gel fraction of 98%), due to the high degree of crosslinking, which resulted in the lowest degradation. The in vitro degradation of the PVA-g-GMA/SF-g-GMA hydrogel showed relatively minimal degradation over 16 weeks. This ensures proper tissue remodeling or regeneration, which the scaffold must degrade at a suitable rate [45]. Meniscal tissue engineering scaffolds should degrade at a minimum pace of 12 months to allow for a proliferation of cells that survive and their replacement of the scaffold [46].

### 3.7. Live and Dead Cells

**Figure 10:**
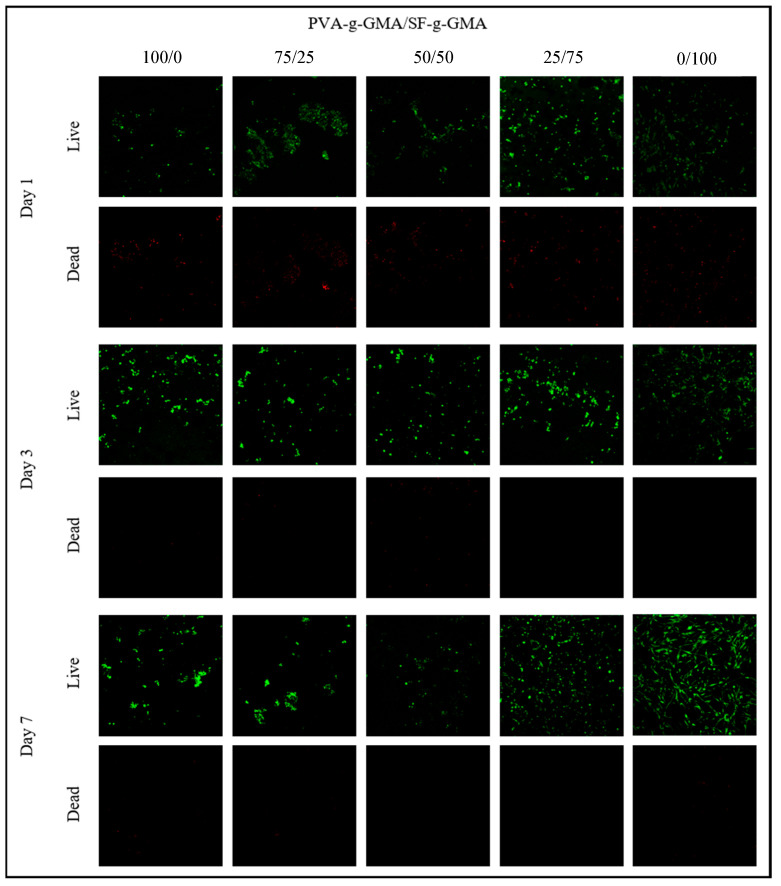
Live–dead staining demonstrated the viability of HCPCs seeded in the PVA-g-GMA/SF-g-GMA biphasic hydrogel on day 1, day 3, and day 7.

The viability of HCPCs seeded in different ratios of PVA-g-GMA/SF-g-GMA biphasic hydrogel on days 1, 3, and 7 was demonstrated using live and dead staining, as shown in Figure 10. Green fluorescence represented live cells, while red fluorescence demonstrated dead cells. Generally, green fluorescence was more predominant in every PVA-g-GMA/SF-g-GMA ratio. The density of live cells increased from day 1 to day 7 in every group except in the pure PVA-g-GMA group, which increased from day 1 to day 3 and then decreased afterward.

**Figure 11:**
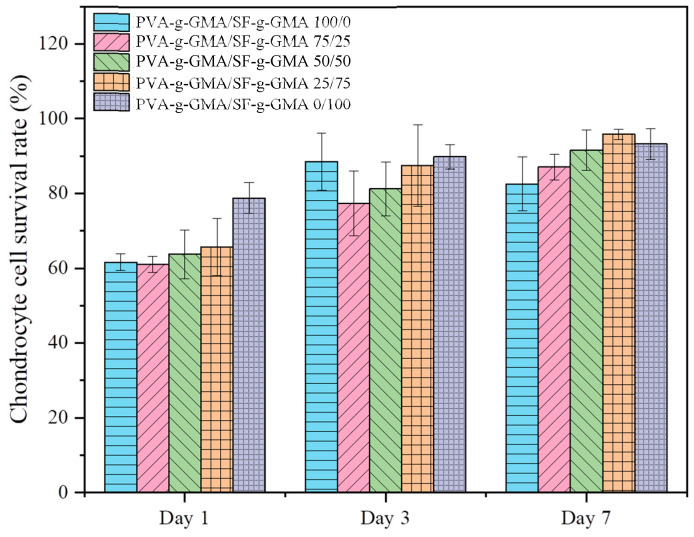
The bar chart displays the survival rates of HCPCs seeded in various ratios of PVA-g-GMA/SF-g-GMA biphasic hydrogel in culture at day 1, 3, and 7.

The viability of HCPCs seeded in biphasic hydrogels mainly increased from day 1 to day 7, as shown in Figure 11. On day 1, the PVA-g-GMA/SF-g-GMA hydrogel at a ratio of 0/100 demonstrated an 80% cell survival rate, while PVA-g-GMA/SF-g-GMA ratios of 100/0, 75/25, 50/50, and 25/75 presented survival rates of 60–65%. This difference could be attributed to the higher biocompatibility of silk fibroin [32]. After 3 days and 7 days, the survival rate of HCPCs in most groups increased, except in the pure PVA-g-GMA hydrogel. Moreover, HCPCs in the pure PVA-g-GMA hydrogel had the lowest survival rate compared to other groups by day 7. In PVA-g-GMA/SF-g-GMA, HCPC vitality increased from Day 1 to Day 7 and exceeded 80%. This confirms that the unreacted macromers were non-toxic to the cells.

These results indicate the advantage of using combined PVA-g-GMA and SF-g-GMA hydrogels, compared to only PVA-g-GMA or SF-g-GMA scaffolds. The viability of the cells after photocrosslinking was in an acceptable range, and the technique was deemed suitable for use in minimally invasive knee surgery.

### 3.8. Gene Expression

**Figure 12:**
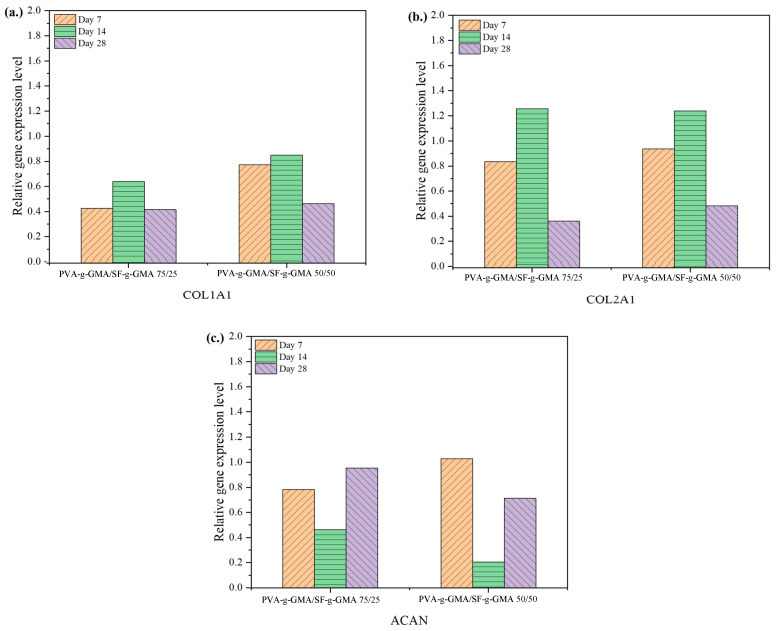
Gene expression of HCPCs in various ratios of PVA-g-GMA/SF-g-GMA biphasic hydrogels was analyzed through the qRT-PCR analysis of (**a**) COL1A1, (**b**) COL2A1, and (**c**) ACAN at days 7, 14, and 28. The data were reported as relative ratios against a housekeeping gene.

According to mechanical properties testing, the 75/25 and 50/50 PVA-g-GMA/SF-g-GMA hydrogels possessed superior compressive moduli. Therefore, these two groups were chosen for seeding HCPCs and further processing. Gene expression analysis of HCPCs was conducted to confirm their chondrogenic phenotypes after seeding in the hydrogels. The expression of Type I collagen (COL1A1) and Type II collagen (COL2A1) in both 75/25 and 50/50 PVA-g-GMA/SF-g-GMA hydrogels increased from day 7 to day 14 and declined by day 28, while a reversed pattern of Aggrecan (ACAN) expression was observed (Figure 12). Comparatively, the 75/25 PVA-g-GMA/SF-g-GMA group demonstrated higher expressions of ACAN at every time point, while the 50/50 PVA-g-GMA/SF-g-GMA hydrogel presented a higher expression of COL1A1 at day 14 and day 28. Regarding COL2A1 expression, the 75/25 PVA-g-GMA/SF-g-GMA hydrogel displayed higher expressions than the other groups at day 7 and day 14, and slightly lower at day 28. These results suggest that PVA-g-GMA/SF-g-GMA, at a ratio of 75/25, supported the chondrogenic phenotype of HCPCs, making it suitable for application in inner meniscus tear lesions. On the other hand, the 50/50 PVA-g-GMA/SF-g-GMA hydrogel promoted less chondrogenicity, resembling the middle area of the native meniscus [3,47].

## 4. Conclusions

In this study, the synthesis of PVA-g-GMA and SF-g-GMA was successfully achieved by grafting with GMA. Various ratios were investigated for forming biphasic injectable hydrogels, by mixing PVA-g-GMA/SF-g-GMA in proportions of 100/0, 75/25, 50/50, 25/75, and 0/100. It was observed that all ratios could be transformed into injectable hydrogels using UV light with a 365 nm wavelength, 6 mW/cm^2^ intensity, and a LAP photoinitiator. The incorporation of GMA into PVA and SF enables the formation of additional crosslinks within the hydrogel matrix. This results in improved mechanical strength, stability, and resistance to degradation, which are desirable properties for tissue engineering scaffolds. The compressive modulus and pore size closely resembled those of the human meniscus. PVA-g-GMA/SF-g-GMA biphasic hydrogels exhibited superior degradation properties compared to unmixed PVA-g-GMA and SF-g-GMA hydrogels.

The HCPCs viability analysis revealed good biocompatibility and non-toxicity, confirming the potential of using PVA-g-GMA/SF-g-GMA hydrogel as a scaffold for meniscus tissue engineering, especially given that HCPCs are representative of human chondrocyte progenitor cells currently used in clinical practice. Additionally, both 75/25 and 50/50 PVA-g-GMA/SF-g-GMA hydrogels were found to promote specific cellular phenotypes, suggesting their potential to serve in area-specific meniscus tear treatment. Further research and preclinical studies are warranted to validate their efficacy and safety for various tissue engineering applications, paving the way for clinical translation and therapeutic use.

## Figures and Tables

**Table 1:** Sequences of primer sets for RT-qPCR [3].

Gene		Primer Sequence (5′ to 3′)
Type I collagen (COL1A2)	Sense	GGA GGA GAG TCA GGA AGG
	Antisense	GCA ACA CAG TTA CAC AAG G
Type II collagen (COL2A1)	Sense	GGC AGA GGT ATA ATG ATA AG
	Antisense	ATG TCG TCG CAG AGG
Aggrecan (ACAN)	Sense	ATA CCG TCG TAG TTC C
	Antisense	TCC TTG TCT CCA TAG C

**Table 2:** The mechanical properties of PVA-g-GMA/SF-g-GMA hydrogel.

PVA-g-GMA/SF-g-GMA	Compressive Modulus (kPa)	Compressive Strength (kPa)
100/0	173.74 ± 58.41	145.57 ± 41.81
75/25	145.92 ± 32.49	100.66 ± 28.75
50/50	117.24 ± 8.29	89.49 ± 13.02
25/75	93.33 ± 15.11	69.55 ± 10.62
0/100	11.03 ± 1.42	53.20 ± 10.81
Human meniscus	100–150	-

## Data Availability

Data are contained within the article.
